# Lewis Y (Le^y^) orchestrates leukocyte trafficking and inflammatory remodeling in leprosy patients from the Brazilian Amazon

**DOI:** 10.1007/s12026-026-09776-0

**Published:** 2026-04-09

**Authors:** Sintia Almeida, Ermelinda do Rosário Moutinho da Cruz, Rosane do Socorro Pompeu de Loiola, Tereza C. O. Corvelo

**Affiliations:** 1 Instituto de Ciências Biológicas - ICB - Universidade Federal do Pará – UFPA, Belém-Pará, Brazil; 2 Secretaria de Estado da Saúde do Pará – SESPA, Belém-Pará, Brazil

**Keywords:** Lewis Y antigen, Glycan remodeling, Leprosy, Endothelial activation, DC-SIGN, Inflammation, Brazilian Amazon

## Abstract

Leprosy is characterized by a complex inflammatory microenvironment in which host-related factors play a decisive role in disease progression. However, the contribution of tissue glycans to inflammatory remodeling remains poorly understood. In this study, we investigated the expression of Lewis blood group antigens in skin biopsies from patients with different clinical forms of leprosy in the Brazilian Amazon, a region marked by persistent hyperendemic transmission. Using immunohistochemistry, we demonstrate that the difucosylated epitope Lewis Y (Le^y^) is preferentially expressed in inflamed lesions, particularly in endothelial cells, histiocytes, lymphocytes, and neural structures, while being absent in healthy skin. Quantitative and spatial analyses revealed that Le^y^ displays the broadest and most intense expression pattern among the investigated Lewis antigens, whereas Lewis X (Le^x^) and sialyl-Lewis x (sLe^x^) exhibited restricted, tissue-dependent expression profiles. Statistical analyses showed that Le^y^ expression varies significantly according to the clinical spectrum of leprosy, while Lewis X and sialyl-Lewis X are primarily dependent on tissue compartment rather than clinical classification. Mechanistically, our findings support a model in which endothelial Le^y^ functions as a central glycan regulator of the inflammatory microenvironment, promoting leukocyte recruitment, endothelial activation, angiogenesis, and immune–vascular interactions. We propose a Le^y^-driven glyco-immune axis, involving DC-SIGN–mediated interactions with dendritic cells and macrophages, as a unifying mechanism linking glycan remodeling, immune cell trafficking, vascular remodeling, and tissue reorganization in leprosy lesions. Together, these results identify Le^y^ as a tissue-specific glycan signature of inflammatory remodeling and highlight glycan remodeling as a previously underexplored organizing principle in leprosy immunopathology, with translational implications for biomarker discovery, risk stratification, and therapeutic targeting in endemic settings.

**Supplementary Information:**

The online version contains supplementary material available at 10.1007/s12026-026-09776-0.

## Introduction

The etiological agent of leprosy, *Mycobacterium leprae*, is transmitted primarily from person to person through respiratory droplets released by untreated multibacillary patients during close and prolonged contact with susceptible individuals. The more intimate and prolonged the contact, the greater the likelihood of infection [1]. Review studies further indicate that transmission remains a major challenge in endemic areas, even in the presence of effective therapeutic regimens [2]. Most individuals exposed to *M. leprae* do not develop clinical disease, reflecting the high level of natural resistance in the human population. Among those who develop disease, the clinical course shows marked heterogeneity [3, 4].

The initial manifestation may evolve toward spontaneous cure or progress to overt clinical disease. Thus, depending on the host immune response, an infected individual may develop one of the disease forms, arranged along a spectrum. At one pole is tuberculoid leprosy (TT), a form associated with high resistance to *M. leprae* infection, characterized by a robust cell-mediated immune response, granulomatous lesions, and few acid-fast bacilli detected in biopsies. At the opposite pole lies lepromatous leprosy (LL), a form of high susceptibility and a multibacillary disease marked by excessive bacillary proliferation and the presence of microcolonies (“globi”) within lesions. This form results from deficient cell-mediated immunity, with dissemination of infection to the viscera and peripheral nerves and a more severe clinical course [5–7]. Between these polar forms are the borderline manifestations including borderline tuberculoid (BT) and borderline lepromatous (BL), which represent immunologically unstable states characterized by intermediate immune responses. Additionally, indeterminate leprosy (IN) is considered an early and undifferentiated form that may either resolve spontaneously or evolve toward one of the defined clinical forms along the spectrum [5–7].

In addition to immunological factors, individual genetic predisposition is known to play a relevant role in susceptibility or resistance to *M. leprae* infection [8, 9]. Several genes and regions of the human genome have been associated with susceptibility to leprosy or with specific clinical forms of the disease [10–18]. These findings suggest that susceptibility to leprosy is multigenic, with a high degree of heterogeneity among different populations [13]. Conversely, the genetic complexity underlying leprosy susceptibility poses substantial challenges for association studies [11].

At the same time, there is evidence that blood group systems are involved in susceptibility or resistance to a variety of infectious and non-infectious diseases [19–21]. Lewis blood group antigens are among the host-related genetic factors that likely regulate pathogen–host interactions, acting as receptors for microorganisms or facilitating adhesion, and correlations have already been described between specific Lewis antigen phenotypes and human diseases [22–24].

Lewis blood group antigens comprise a heterogeneous family of oligosaccharides sharing a common structural backbone (Galβ1-3GlcNAc-R, type I, or Galβ1-4GlcNAc-R, type II), to which α(1,3)-linked fucose residues are added. This results in the formation of Lewis A/X (Le^a/x^) antigens, which are fucosylated trisaccharides derived from type I and type II precursor chains, respectively. In contrast, Lewis B/Y antigens represent the corresponding difucosylated derivatives of Lewis A/X, with the distinction from Lewis A/X antigens being solely the addition of a terminal α(1,2)-linked fucose residue [25].

Similarly, the sialyl-Le^a^ and sialyl-Le^x^ antigens are generated by the addition of a sialic acid residue through an α(2,3) linkage, followed by the incorporation of a fucose residue via an α(1,4) or α(1,3) linkage on type I or type II precursor chains, respectively. The human epidermis expresses Lewis histo-blood group antigens exclusively in the form of type II chains [26, 27].

In this context, it becomes essential to evaluate the potential association between host genetic markers of exposure, such as Lewis system antigens expressed on epithelial cells and in secretions, which participate in cellular recognition and communication processes, leukocyte trafficking, and inflammatory responses, and the mechanisms underlying *M. leprae* infection.

Considering that the clinical manifestations resulting from *M. leprae* infection are strongly influenced by the host’s hereditary predisposition and given that leprosy remains an endemic infectious disease in several municipalities of the state of Pará, this study investigated the expression of Lewis blood group antigens through histopathological and immunohistochemical analyses of skin lesion biopsies from individuals affected by different clinical forms of leprosy. The aim was to investigate their potential association with the mechanisms underlying this infection.

The persistence of leprosy as a public health problem in Amazon regions, where transmission remains active and heterogeneous even after decades of control policies, underscores the complexity of disease elimination efforts [2]. Evidence indicates that the clinical variability of leprosy is strongly dependent on host genetic factors, including antigens of the Lewis family, which modulate inflammation, cell adhesion, and interactions with receptors such as DC-SIGN [10, 19]. Therefore, characterizing the expression of Lewis antigens in affected tissues remains relevant not only for elucidating underexplored immunopathological pathways but also for supporting contemporary strategies for surveillance, early diagnosis, and risk stratification in hyperendemic Amazonian regions.

## Methodology

### Study design and population

#### Study design

This was a descriptive, observational, cross-sectional, and retrospective study.

#### Study population

A total of 100 skin lesion biopsy samples with clinical and diagnostic suspicion of leprosy were analyzed. These samples were referred to the Histopathology Service of the Central Public Health Laboratory of the State of Pará. The distribution of clinical forms was as follows: 46 cases (46%) corresponded to the indeterminate form (IN), 36 (36%) to the tuberculoid form (TT), 3 (3%) to the lepromatous form (LL), 12 (12%) to borderline tuberculoid (BT), and 3 (3%) to borderline lepromatous (BL). Normal human skin specimens were used as controls to evaluate the expression of Lewis antigens in this tissue. Positive controls consisted of 21 samples from patients with tuberculosis, used to validate the immunohistochemical detection of mycobacterial antigens, in which immunoreactivity of macrophages and other epidermal structures was observed. In all cases, negative controls were included by replacing the primary anti-*Mycobacterium tuberculosis* antibody with a bovine serum albumin solution, resulting in the absence of staining in these negative control samples.

### Ethical approval

The study was approved by the Research Ethics Committee of the Institute of Health Sciences, Federal University of Pará (UFPA), in accordance with Resolution No. 196/96 of the Brazilian National Research Ethics Council.

### Epidemiological indicators

#### Leprosy detection rate

The detection rate was calculated according to the criteria established by the Brazilian Ministry of Health, based on the ratio between the number of confirmed cases and the total number of inhabitants, multiplied by 10,000 [28]. Accordingly, leprosy detection rates per 10,000 inhabitants were classified as low (less than 0.2), medium (0.2–0.9), high (1.0–1.9), very high (2.0–3.9), and hyperendemic (greater than or equal to 4.0).

### Histopathological analysis

Routine hematoxylin–eosin (H&E) staining was performed for histopathological diagnosis and classification of leprosy according to the criteria of Ridley and Jopling (1966) [6], in addition, Fite–Faraco staining [29] was carried out for the detection of Hansen’s bacillus in skin biopsy specimens.

### Immunohistochemistry

#### Sample processing and staining protocol

Formalin-fixed skin fragments were processed, embedded in paraffin blocks, serially sectioned at a thickness of 4–5 μm, and mounted on silanized slides. These sections were subjected to an indirect immunohistochemical technique (adapted from Pedal, 1987; Pedal et al., 1989) for the detection of Le^y^, Le^x^, sLe^a^, sLe^x^, Le^a^, Le^b^ antigens [30, 31], following standard immunohistochemical procedures for formalin-fixed paraffin-embedded tissues as previously described [32, 33].

Initially, the slides were heated in an oven and deparaffinized in xylene. For antigen detection, the slides were rehydrated through graded ethanol baths of decreasing concentrations and washed twice for 5 min in Tris/HCl buffer containing Triton X-100 (pH 7.4). The sections were then incubated in 15% acetic acid for 10 min to block endogenous alkaline phosphatase activity, followed by additional washing in Tris/HCl/Triton buffer. All sections tested for antigen expression were incubated in blocking buffer (Tris/HCl buffer with bovine serum albumin at a 1:20 dilution), followed by incubation with primary monoclonal antibodies against Le^y^, Le^x^, sLe^a^, sLe^x^ Le^a^, Le^b^, as well as *M. leprae*. For the latter, a cross-reactive reaction was performed to detect mycobacterial antigens using antibodies raised against *M. tuberculosis*.

Subsequently, the test slides were incubated again in blocking buffer and then incubated for 12 h at 4 °C with an alkaline phosphatase–conjugated anti-mouse (IgM) secondary antibody (1:1000 dilution). Color development was carried out using the Histomark Red kit (KPL Laboratories) for 20–30 min, according to the manufacturer’s instructions. The sections were then thoroughly washed in distilled water, counterstained with hematoxylin, dehydrated in ethanol, and mounted with synthetic resin and coverslips.

#### Immunohistochemical controls

##### **Negative** control

 Primary and secondary antibodies were replaced with Tris/saline buffer in the reaction.

##### Positive control

Internal cellular structures known to express the antigens under investigation were used as positive controls.

#### Quantification and scoring

The staining pattern for Lewis antigens in the skin (epidermis and dermis, including vessels and glands) was classified according to an ordinal method based on the type of staining observed, using a semiquantitative evaluation. Reactions were considered positive when homogeneous staining was observed throughout the entire analyzed region. Reactions were grouped as heterogeneous patterns when both stained and unstained cells were present, and as negative patterns when no staining was detected. Antigen expression was quantified using *H-*score criteria, a variation of the immunoreactivity score (IRS), according to the protocol described by Meyerholz and Beck (2018) [34]. The *H-score* was calculated as: H-score = Σ (Pi × i), where Pi represents the percentage of cells stained at each intensity level (0–3), resulting in a score ranging from 0 to 300. All slides were independently evaluated by two observers blinded to the clinical classification (Table 1).

**Table 1:** Specific antibodies for genetic predisposition markers to *M. leprae* infection: Lewis antigens

Antibodies	Dilution	Clone	Reference
Anti-Le^x^	1:100	C3D-1	DAKO/Agilent
Anti- Le^y^	1:50	A70-C/C8.	Santa Cruz Biotechnology
Anti-Le^a^	1:100	Seraclone Anti-(LE1)	Biotest
Anti-Le^b^	1:100	Seraclone Anti-(LE2)	Biotest
Anti-sialyl-Le^a^	1:50	116-NS-19-9	DAKO/Agilent
Anti-sialyl-Le^x^	1:50	CSLEX1	BD Biosciences

### Statistical analysis

The relationship between the expression of the molecules investigated and the histopathological variables of leprosy was analyzed using appropriate statistical tests to detect differences between the study groups. These included the chi-square test (χ²) or G-test (Woolf), the Kruskal–Wallis test, and the binomial test, with statistical significance accepted at the 95% confidence level. Analyses were performed using BioEstat 5.0 software (Ayres et al., 2007), and figures were created using the Python programming language and its libraries, including matplotlib [35], pandas (https://github.com/pandas-dev/pandas), scipy [36], numpy [37], seaborn [38], and sklearn (https://scikit-learn.org/stable/getting_started.html).

## Results

### Epidemiology

**Fig. 1:**
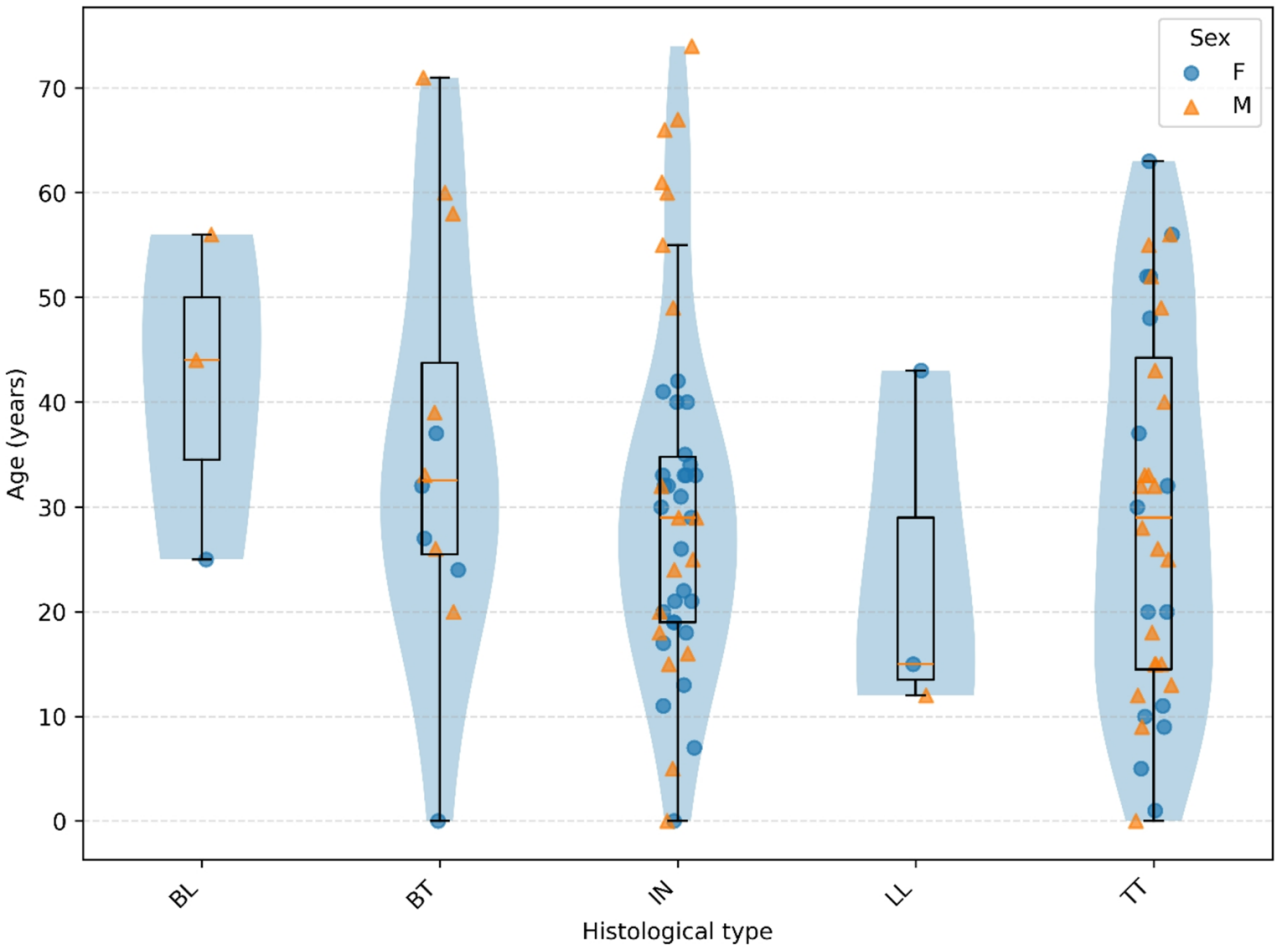
Age distribution across histopathological forms of leprosy stratified by sex. Raincloud plot showing the age distribution of patients across histopathological classifications: borderline lepromatous (BL), borderline tuberculoid (BT), indeterminate (IN), lepromatous (LL), and tuberculoid (TT). Violin plots represent the distribution density within each group. Overlaid boxplots indicate the median (central line), interquartile range (boxes), and data dispersion. Individual observations are displayed as jittered points to reduce overlap and are colored according to sex (F: female; M: male). This visualization enables simultaneous assessment of distribution, central tendency, and variability across clinical forms

A total of 100 skin biopsies with a diagnosis of leprosy were evaluated: 46 (46%) corresponded to the indeterminate form (IN), 36 (36%) to the tuberculoid form (TT), 3 (3%) to the lepromatous form (LL), 12 (12%) to borderline tuberculoid (BT), and 3 (3%) to borderline lepromatous (BL). The mean age was 31 years, ranging from 6 months to 74 years. The distribution of clinical forms by sex was similar, with no statistically significant difference observed (Fig. 1).

### Spatial heterogeneity of leprosy detection rates in the State of Pará

**Fig. 2:**
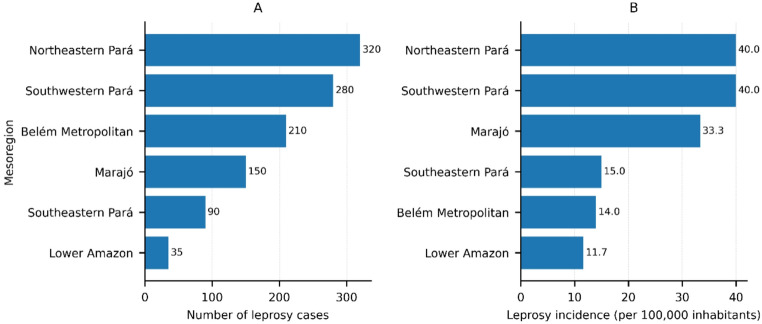
Leprosy burden across mesoregions of Pará State, Brazil. (**A**) Number of reported leprosy cases by mesoregion. Bars are ordered from the highest to the lowest number of cases. (**B**) Leprosy incidence expressed as cases per 100,000 inhabitants in each mesoregion. Bars are ordered from the highest to the lowest incidence, allowing direct visual comparison of relative burden across regions

Leprosy in the state of Pará exhibits a heterogeneous geographic distribution, characterized by the coexistence of areas with low disease occurrence and regions with a high concentration of cases. As illustrated in Fig. 2, municipalities in the Northeast mesoregion account for more than 30% of all cases reported in the state, constituting one of the main endemic burden areas.

Although no direct association was observed between region or municipality and the different clinical forms of leprosy, it is noteworthy that the Northeast mesoregion concentrates 67% of LL cases, in addition to all cases classified as BL. This pattern suggests an epidemiological context marked by greater clinical severity and reinforces the relevance of this region within the leprosy landscape in Pará.

**Fig. 3:**
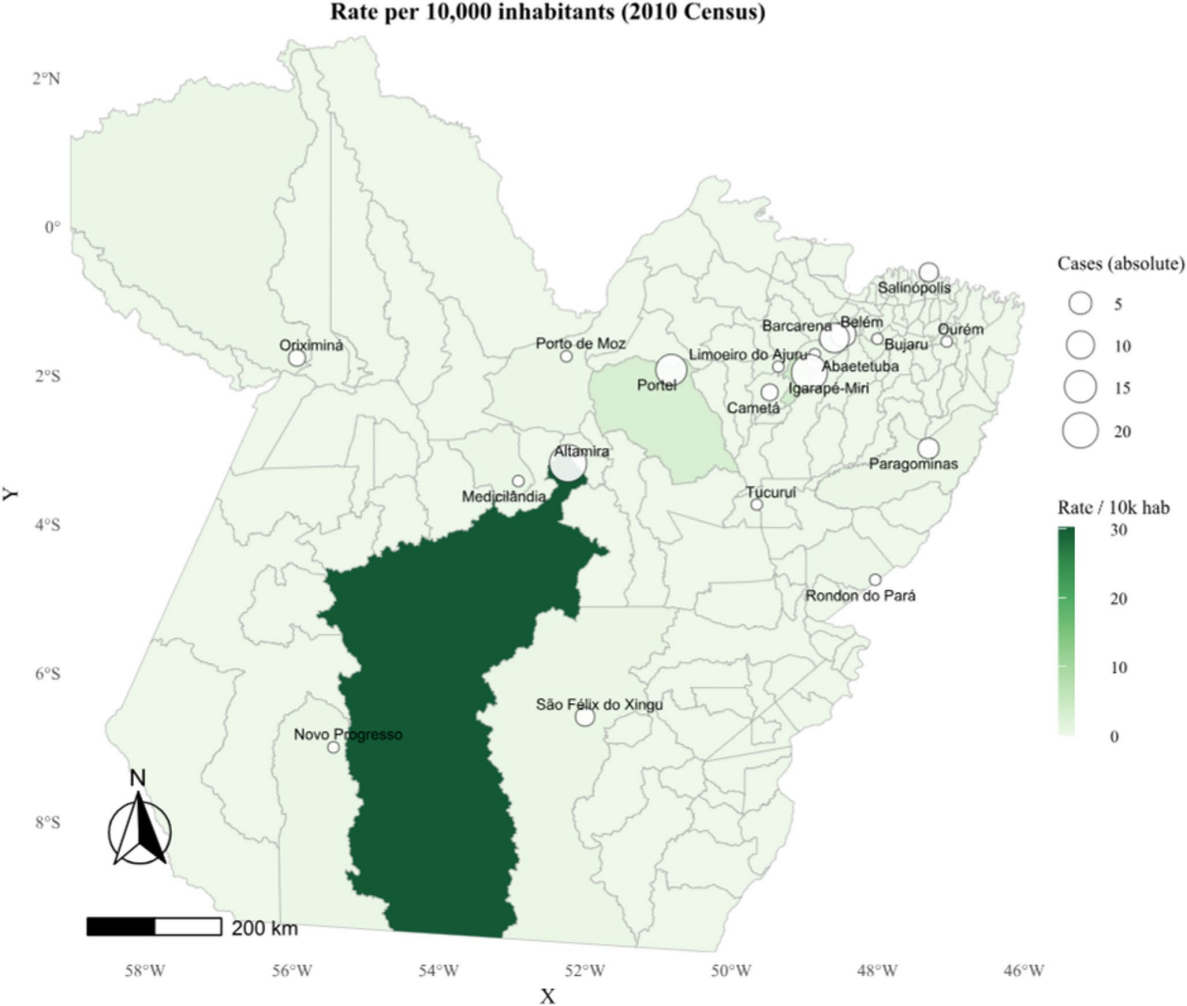
Leprosy detection rate per 10,000 inhabitants (2010 Census) in the municipalities of the state of Pará. The figure highlights the spatial heterogeneity of the endemic distribution, with higher-risk areas concentrated in the Southwest and Northeast regions of the state. This information contextualizes the epidemiological environment in which the samples analyzed in this study were obtained, reinforcing the relevance of investigating the mechanisms underlying Lewis antigen expression associated with the local inflammatory microenvironment

Figure 3 complements this analysis by presenting the leprosy detection rate per 10,000 inhabitants, based on the 2010 Census, revealing territorial clusters of higher endemicity, particularly in the Northeast and Southwest regions of the state. In contrast, areas in the central and western portions exhibit lower detection rates, further reinforcing the uneven distribution of the disease across the territory of Pará.

This spatial pattern reflects persistent conditions of hyperendemic transmission, especially in regions where the disease has remained active over time. This scenario is particularly relevant considering that the samples analyzed in this study were obtained within a context of prolonged transmission, characteristic of the Brazilian Amazon.

### Histopathological diagnosis and immunohistochemical detection of *Mycobacterium* spp

In the present study, bacilli were detected in skin biopsies by the Fite–Faraco staining assay in only 3% of the cases. In contrast, immunohistochemical analysis identified acid-fast bacillary fragments in 90% of skin biopsies from patients with leprosy. In most of these samples, bacilli had not been identified by conventional bacteriological evaluation using Fite–Faraco staining.

In this immunohistochemical analysis, antigenic labeling of bacillary fragments was detected in multiple epidermal compartments, predominantly in histiocytes, and more rarely in sweat glands and endothelial cells.

The immunohistochemical findings in skin biopsies from leprosy patients using the anti-*M. tuberculosis* marker are summarized in Table 2. This assay detected histiocyte staining in all patients classified as BT, BL, and LL. Staining intensity was classified as negative (no staining), weak, moderate, or strong, based on the proportion and intensity of immunoreactive cells.

**Table 2:** Immunohistochemical evaluation using anti-M. tuberculosis antibody in skin biopsies from individuals with a histopathological diagnosis of leprosy, Pará, 2000–2008

Clinical form	Area	Negative	Weak	Moderate	Strong
BT (*N* = 12)	Histiocytes	0	7	3	2
	Sweat glands	8	2	1	1
	Endothelium	12	0	0	0
BL (*N* = 3)	Histiocytes	0	0	1	2
	Sweat glands	2	0	1	0
	Endothelium	3	0	0	0
IN (*N* = 46)	Histiocytes	11	21	8	6
	Sweat glands	36	8	2	0
	Endothelium	45	0	1	0
TT (*N* = 36)	Histiocytes	8	17	5	6
	Sweat glands	24	7	1	4
	Endothelium	34	1	0	1
LL (*N* = 3)	Histiocytes	0	0	0	3
	Sweat glands	3	0	0	0
	Endothelium	3	0	0	0
Total	Histiocytes	19	45	17	19
	Sweat glands	73	17	5	5
	Endothelium	97	1	1	1

This polyclonal anti-*M. tuberculosis* antibody did not react with skin biopsies from healthy control individuals, nor with samples from individuals affected by other diseases.

### Lewis antigens

With respect to Lewis family antigens, epidermal expression is not regulated by the secretor gene (FUT2) but rather depends on the H gene (FUT1).

**Fig. 4:**
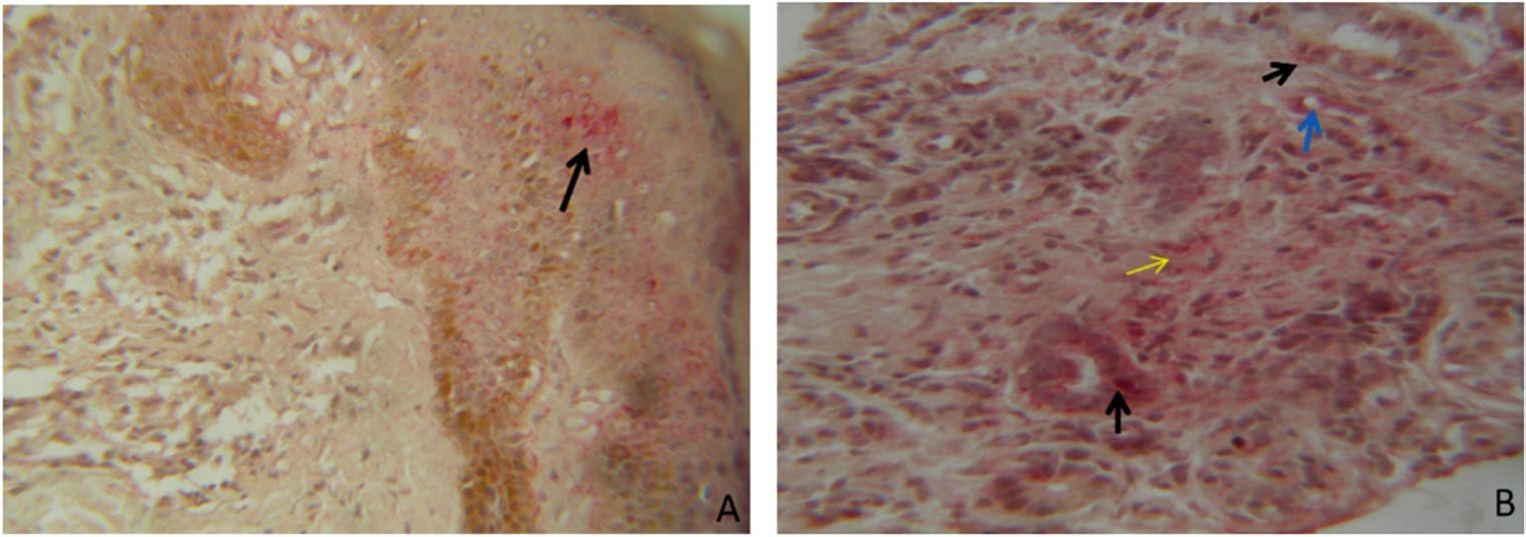
Reaction pattern for the Lewis Y antigen. (**A**) Heterogeneous positivity in the epidermis (thin arrow). (**B**) Positivity in sweat glands (black arrow), vascular endothelial cells (blue arrow), and inflammatory cells within the lesion (yellow arrow). Immunohistochemical method using alkaline phosphatase. Counterstain: hematoxylin. Magnification: 400×

The Lewis Y antigen (Le^y^) was detected in 46% of individuals with leprosy. This antigen was observed either in isolation or in combination in different epidermal compartments, including vascular endothelium, histiocytes, lymphocytes, epithelioid cells, and sweat glands (Fig. 4). In contrast, its expression in neutrophils and nerve tissue was detected at very low frequency.

In the control group, consisting of normal skin from healthy individuals, the antibody against Le^y^ did not stain any of these histological structures (0% positivity). Accordingly, representative images of control tissue were not included in Fig. 4, as no immunoreactivity was observed in these samples. This is because the expression of the Le^y^ antigen needs to be induced by immunological mediators, as occurs during inflammatory processes. This absence reflects that Le^y^ expression is not constitutive in normal skin but is induced by immunological mediators during inflammatory processes.

At the cellular level, immunohistochemical staining for Le^y^ showed a predominance of cytoplasmic labeling, frequently associated with concurrent membrane activity, including apical membrane staining of the cytoplasmic membrane in some regions, particularly evident in glandular structures.

**Fig. 5:**
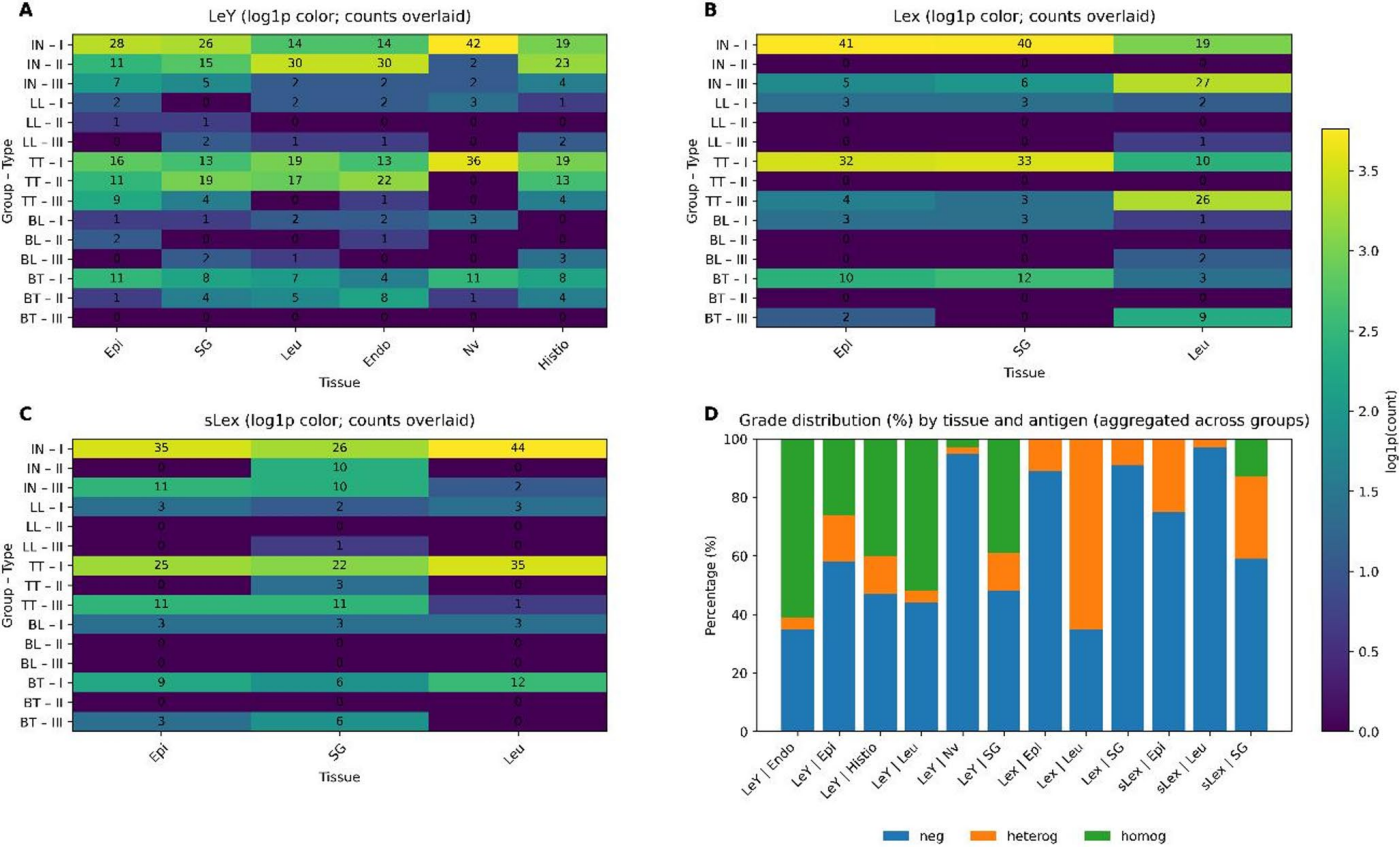
Differential expression patterns of Lewis antigens (Le^y^, Le^x^ and sLe^x^) across tissues and clinical forms of leprosy. Panels **A**–**C**: Heatmaps showing the distribution of antigen expression counts in epidermis (Epi), sweat glands (SG), leukocytes (Leu), endothelial cells (Endo), nerve fibers (Nv), and histiocytes (Histio) according to clinical form of leprosy. Panel **A**: Le^y^; Panel **B**: Le^x^; Panel **C**: sLe^x^. Colors represent log1p-transformed counts, and overlaid numbers correspond to raw observed counts. Rows indicate combinations of clinical form and expression degree: (I) negative, (II) positive homogeneous, and (III) positive heterogeneous. Clinical forms are defined as indeterminate (IN), tuberculoid (TT), borderline tuberculoid (BT), borderline lepromatous (BL), and lepromatous (LL). Panel **D**: Stacked bar plot showing the percentage distribution of antigen expression degrees across tissues and antigens, aggregated by clinical form. Expression degrees are defined as negative (neg; degree I – no staining), heterogeneous (heterog; degree III – staining in 10–90% of cells), and homogeneous (homog; degree II – staining in > 90% of cells). The plot highlights differences in expression profiles of Le^y^, Le^x^, and sLe^x^ across tissues and clinical forms

The distribution of positivity for Lewis system antigens across the different histological structures of the skin and among the distinct clinical forms of leprosy is shown in Fig. 5. Comparative quantitative analysis demonstrated that the Le^y^ antigen exhibited the broadest and most intense expression pattern among the three antigens evaluated, displaying higher absolute counts and wider distribution across the different tissues analyzed. This pattern is clearly illustrated in the heatmaps (Panel A), in which Le^y^ showed higher expression levels in multiple structures, including epidermis, sweat glands, leukocytes, endothelial cells, nerve fibers, and histiocytes. In contrast, the Le^x^ and sLe^x^ antigens exhibited more restricted and less intense expression profiles, as shown in Panels B and C.

The Le^X^ antigen was absent in most skin samples in the epidermis from patients with leprosy. However, some specimens exhibited staining in lesional sites, at low intensity and in variable proportions, on the membranes of cells in the spinous and granular layers, in hair follicles, and in the ducts of sweat glands (Supplementary Figure S1). In addition, a substantial proportion of cases, approximately 65%, expressed the Le^x^ epitope in a variable number of leukocytes; some of the Le^x^-reactive cells displayed morphological features suggestive of macrophages and dendritic cells.

The sLe^x^ epitope was detected on the membranes of cells in the spinous layer of the epidermis, in hair follicles, and in the epithelium of sweat gland ducts, with variable percentages of stained cells and reaction intensity (Supplementary Figure S2). This expression pattern differed between lesional and non-lesional skin areas. In the epithelium of sweat gland ducts within lesional skin, anti–sLe^x^ did not show reactivity. In contrast, reactivity was observed in other affected and inflamed areas (granulomas). Likewise, in non-lesional skin, cells on the surface of the spinous layer, hair follicles, and the epithelium of sweat gland ducts showed positivity.

Exceptionally, in some cases, a small number of leukocytes, especially neutrophils and macrophages, and some endothelial cells were also labeled with the anti-sLe^x^ antibody.

In the control group, the antibody against sLe^x^ stained the stratified epithelium of normal skin with variable intensity in 70% of the samples. The spinous layer was reactive, but positivity was rarely detected in other skin layers. Strong staining was frequently observed in hair follicles, as well as in the apical region of epithelial cells of sweat gland ducts. However, endothelial cells, leukocytes, and neutrophils did not react with the anti-sLe^x^ antibody.

To quantitatively assess the existence of associations among the analyzed variables, chi-square tests of independence were performed using the observed frequencies for each antigen (Table 3). However, the lepromatous and borderline lepromatous forms were excluded from this analysis due to the small number of cases, which precluded reliable statistical testing. Initially, the association between clinical type and degree of expression was investigated. A statistically significant association was observed only for the Le^y^ antigen (χ² = 41.21; *p* < 0.000001), indicating that the intensity and pattern of expression of this antigen vary according to the clinical spectrum of leprosy. For the Le^x^ and sLe^x^ antigens, no significant association was detected between clinical type and degree of expression (χ² = 1.87; *p* = 0.759 and χ² = 9.90; *p* = 0.272, respectively), demonstrating that their expression profiles are relatively independent of clinical type.

Additionally, the association between tissue type and degree of expression was evaluated for each antigen. In this analysis, statistically significant associations were observed for all investigated markers: Le^y^ (χ² = 118.59; *p* < 0.0001), Le^x^ (χ² = 99.41; *p* < 0.0001), and sLe^x^ (χ² = 55.42; *p* < 0.0001). These results demonstrate that the degree of expression of Lewis system antigens is strongly dependent on the type of tissue analyzed, reflecting distinct reactivity patterns among the epidermis, sweat glands, leukocytes, endothelium, nerve fibers, and histiocytes.

**Table 3:** Statistical associations involving the degree of expression of Lewis system antigens

Antigen	Association evaluated	χ²	*p*-value	Significance
Le^y^	Clinical type × Degree	41.21	< 0.000001	Significant
Le^x^	Clinical type × Degree	1.87	0.759	Not significant
sLe^x^	Clinical type × Degree	9.90	0.272	Not significant
Le^y^	Tissue × Degree	118.59	< 0.0001	Significant
Le^x^	Tissue × Degree	99.41	< 0.0001	Significant
sLe^x^	Tissue × Degree	55.42	< 0.0001	Significant

The integrated analysis presented in Panel D of Fig. 5 showed that the Le^y^ antigen displayed a higher proportion of positivity with variable degrees of homogeneous and heterogeneous expression, particularly in the clinical types IN and TT. In contrast, the Le^x^ antigen showed a predominance of negative grading across most combinations of clinical type and specific tissue, whereas sLe^x^ exhibited a more heterogeneous pattern with lower overall intensity, without a consistent trend associated with any specific clinical type.

From a quantitative perspective, the heatmaps suggest that Le^y^ is the antigen with the greatest diversity of positive tissues and the highest frequencies of expression, whereas Le^x^ exhibited predominantly low values, particularly in the epidermis and sweat glands. sLe^x^ displayed an intermediate pattern, with detectable expression in some combinations of clinical type and specific tissue, but without a statistically significant association with the clinical spectrum of the disease.

In this context, the Le^y^ antigen stands out as the only marker whose expression varies significantly according to clinical type, in addition to showing a strong dependence on the type of tissue analyzed. In contrast, Le^x^ and sLe^x^ display expression patterns that are predominantly tissue-dependent but independent of the clinical spectrum, suggesting distinct biological roles in the immunopathogenesis of the disease.

Additionally, the antigens Le^a^, Le^b^ and sialyl-Le^a^ were investigated in epidermal structures but were not detected using the monoclonal antibodies anti-Le^a^, anti-Le^b^, and CA19-9 (anti-sLe^a^), with the exception of anti-Le^b^, which showed reactivity restricted to the epithelium of sweat gland ducts. Because type I chain structures are found only in the epithelium of sweat gland ducts in the skin, a systematic evaluation of differences in expression patterns of these antigens was not feasible. Moreover, we considered that reliable analysis of these data is difficult due to the genetic heterogeneity of these blood group systems in human populations, which would require genotypic and phenotypic determination in blood and saliva for proper comparison and confirmation of the findings.

**Fig. 6:**
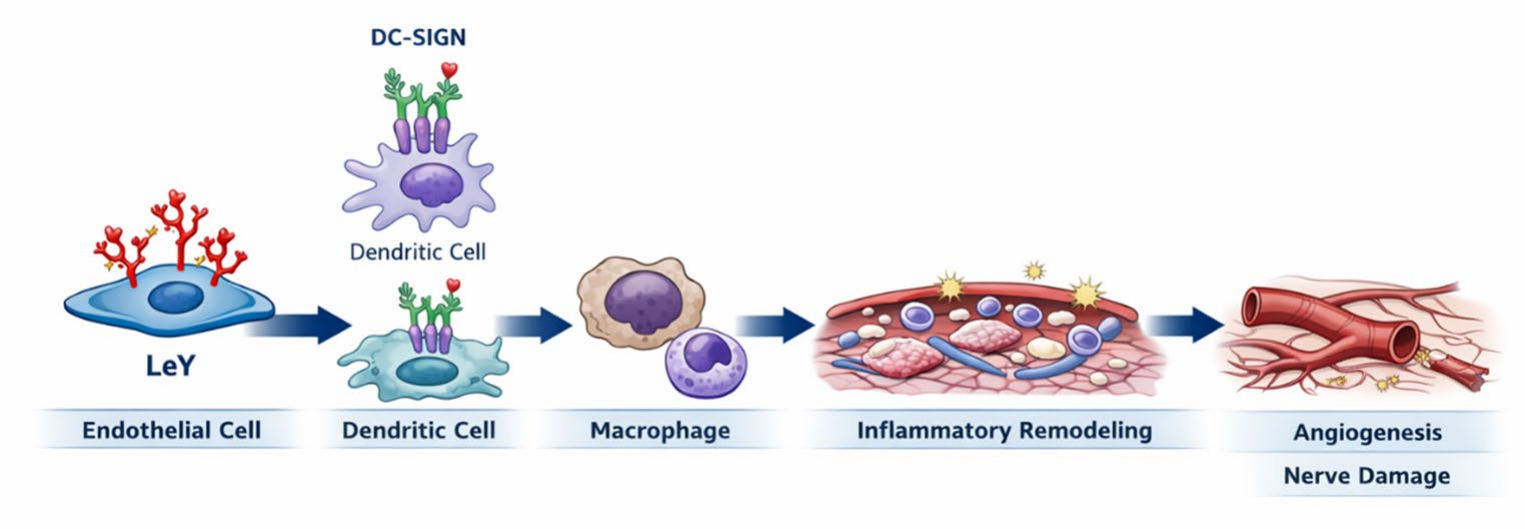
Proposed mechanistic model of Le^y^-driven inflammatory remodeling in leprosy. Endothelial expression of Lewis Y (Le^y^) promotes immune–vascular interactions through DC-SIGN recognition on dendritic cells and macrophages, facilitating leukocyte recruitment, inflammatory remodeling, angiogenesis, and neural damage. This glyco-immune axis integrates endothelial activation, immune cell trafficking, and tissue reorganization in leprosy lesions. (Created using AI-assisted tools)

An integrative analysis of the spatial, cellular, and molecular distribution of Lewis antigens supports a coordinated biological organization of the inflammatory microenvironment. In particular, the preferential endothelial expression of Le^y^, its association with immune cells, and its tissue-specific distribution patterns converge toward a unified mechanistic framework linking endothelial activation, immune cell recruitment, and tissue remodeling. This integrated model is schematically summarized in Fig. 6.

## Discussion

In this study, we investigated the immunohistochemical expression of Lewis family antigens in the epidermis and dermal structures of patients with different clinical forms of leprosy. We observed that glycosylation patterns are altered in inflamed cutaneous areas compared with histologically normal skin, with particular emphasis on the preferential expression of the Lewis Y (Le^y^) epitope in endothelial cells, histiocytes, and lymphocytes within lesional regions. In contrast, non-lesional skin from the same patients exhibited an expression pattern similar to that observed in individuals without leprosy.

Notably, no Le^y^ immunoreactivity was observed in healthy skin. This finding is consistent with the regulation of fucosylated glycans as inducible structures associated with inflammatory activation. Pro-inflammatory mediators are known to modulate glycosyltransferase activity and promote the expression of specific glycan epitopes, including α(1,2)-fucosylated structures, in activated endothelial and immune cells [39]. In this context, α(1,2)-linked fucosylation has been shown to be upregulated in inflamed tissues, where it contributes to leukocyte recruitment and angiogenesis [40]. Image resolution may limit the precise visualization of subcellular localization; however, staining patterns were consistently interpretable across samples.

These findings indicate that alterations in glycan epitope presentation accompany the local inflammatory process and may modulate cell–cell interactions and leukocyte trafficking. To our knowledge, no previous studies have investigated the tissue-specific expression of Lewis antigens in leprosy lesions, which limits direct comparisons. However, our findings can be interpreted in light of mechanistic and glycobiological evidence from other infectious and inflammatory conditions, where glycan-mediated mechanisms regulate immune cell trafficking and host–pathogen interactions. In particular, glycobiological studies indicate that fucosylated glycans act as dynamic regulators of immune recognition through interactions with lectin-like receptors, including DC-SIGN and related C-type lectins, which are central to pathogen recognition and immune response modulation [41, 42].

The spatial heterogeneity observed across the studied regions likely reflects contextual factors beyond the tissue-level mechanisms investigated here. Socioeconomic conditions, including poverty and limited access to healthcare, are known to influence leprosy transmission and persistence [43]. In addition, host genetic background contributes to disease susceptibility, with evidence of strong genetic effects in hyperendemic populations from the Brazilian Amazon [44] and in isolated settings where genetic susceptibility may be enriched [45]. These factors likely provide a broader epidemiological context for the observed patterns, complementing the tissue-level mechanisms explored in this study.

Together, these findings support a mechanistic model in which Le^y^ acts as a central glycan regulator of the inflammatory microenvironment in leprosy. Endothelial Le^y^ expression appears to promote leukocyte recruitment, DC-SIGN–mediated immune interactions, vascular remodeling, and local angiogenesis, thereby orchestrating tissue inflammation and structural reorganization. This glyco-immune axis provides a unifying framework linking glycan remodeling, immune cell trafficking, endothelial activation, and tissue damage in leprosy lesions. Rather than acting as passive structural components, glycans are increasingly recognized as active organizers of immune microenvironments, capable of integrating inflammatory signals with cellular positioning and activation states [42, 46].

The preferential expression of Le^y^ in endothelial cells is consistent with studies indicating that Lewis epitopes are regulated by pro-inflammatory signals (e.g., IL-1β and TNF-α) and can be mobilized from the cytoplasm to the plasma membrane, thereby altering endothelial function in terms of adhesion, signaling, and angiogenesis. These properties support the role of Le^y^ as a modulator of leukocyte recruitment and local vascular responses in infectious and inflammatory processes, further supporting its biological relevance plausible [26, 47–49]. In parallel, the regulation of Lewis antigen expression through fucosyltransferases (FUT2 and FUT3) provides a molecular link between inflammatory signaling and glycan remodeling, suggesting that host genetic variation may influence susceptibility and disease progression in infections such as leprosy [50–52].

From a molecular perspective, C-type lectins, most notably DC-SIGN (CD209) and related lectins, recognize oligosaccharides containing fucose residues, including Lewis epitopes (Le^x^, Le^y^, Le^a^, Le^b^). The interaction between endogenous ligands expressing Le^y^ on endothelial cells/ICAMs and receptors such as DC-SIGN on dendritic cells and macrophages provides a plausible mechanism for facilitating transendothelial migration, antigen capture, and immune modulation at the site of *M. leprae* infection. In experimental models and studies on mycobacteria, the association between DC-SIGN and *M. leprae* (mediated by bacterial glycoconjugates such as Man-LAM) has been linked to immune response polarization, potentially favoring tolerance or inflammation depending on the signaling context [53–55]. This is consistent with broader evidence showing that mycobacterial glycans exploit lectin receptors to modulate host immunity and promote intracellular survival, highlighting a conserved strategy among pathogens to hijack glycan-recognition pathways [41, 42].

Literature also supports a functional role for Le^y^ in angiogenesis. Studies using recombinant lectin domains (e.g., from thrombomodulin) and endothelial assays have demonstrated that Le^y^ participates in chemotaxis and vessel formation, and that its interaction with specific ligands can modulate these processes. Accordingly, the strong Le^y^ staining observed in the endothelium of biopsies may be associated not only with enhanced inflammatory recruitment but also with local angiogenic alterations that influence the microarchitecture of leprosy lesions [56]. Such vascular remodeling is increasingly recognized as an integral component of chronic inflammatory responses, where glycan-dependent mechanisms contribute to endothelial activation and tissue restructuring [57].

An additional relevant finding was the intense staining of the Le^y^ antigen in neural structures, particularly in the IN and TT forms. Considering that leprosy is classically characterized by perineural inflammation and progressive damage to peripheral nerves, the expression of Le^y^ in these structures suggests that alterations in the neural and perineural glycocalyx may be part of the mechanisms involved in leprosy-associated neuropathy. The presence of Le^y^ in nerves may reflect both endothelial and inflammatory activation of the perineural microenvironment [58, 59], as well as tissue remodeling processes associated with chronic inflammation, thereby contributing to the neural dysfunction observed clinically. This interpretation is further supported by evidence that immune-mediated mechanisms, rather than direct bacterial toxicity alone, are major drivers of nerve damage in leprosy [3, 60].

Statistical analysis further supports these interpretations. A highly significant association between clinical type and degree of expression was observed exclusively for the Le^y^ antigen (χ² = 41.21; *p* < 0.000001), demonstrating that the intensity of its expression varies according to the clinical spectrum of leprosy. In contrast, no association was identified between clinical type and degree of expression for Le^x^ (*p* = 0.759) and sLe^x^ (*p* = 0.272), indicating that these epitopes behave independently of the clinical classification of the disease.

A statistically significant association was found between tissue type and degree of expression for all analyzed antigens, Le^y^ (χ² = 118.59; *p* < 0.0001), Le^x^ (χ² = 99.41; *p* < 0.0001), and sLe^x^ (χ² = 55.42; *p* < 0.0001), demonstrating that the expression pattern of the Lewis system is strongly dependent on the tissue compartment. These results explain the heterogeneity observed in the heatmaps and indicate that different tissues exhibit specific glycosylation profiles during the leprosy-associated inflammatory process. Such compartmentalized regulation of glycosylation is consistent with current models in which epithelial, endothelial, and immune cells exhibit distinct glycan signatures in response to local inflammatory cues [1, 41, 42, 61].

Beyond Le^y^, the Le^x^ and sLe^x^ epitopes exhibited more restricted and heterogeneous expression patterns, predominantly in leukocytes and, occasionally, in endothelial cells and glandular epithelium. Although they did not show statistically significant associations with the clinical subtypes of leprosy, these findings are biologically consistent with the classical roles of Le^x^ (CD15) and sLe^x^ as functional ligands for selectins. Le^x^ is widely implicated in processes of cell–cell adhesion and leukocyte activation, whereas sLe^x^ constitutes the main ligand of E-selectin and is essential for leukocyte rolling and cellular extravasation in inflamed tissues [62, 63]. Accordingly, their expression patterns likely reflect dynamic leukocyte recruitment processes rather than disease-specific structural alterations. Representative supplementary staining patterns for Lex and sLex are provided in Supplementary Figures S1 and S2.

In the present study, the Le^x^ antigen showed predominant expression in leukocytes and only focal, low intensity staining in epithelial and endothelial cells, with no statistically significant association with the histological types of leprosy, as determined by chi-square analysis (χ² = 1.87; *p* = 0.759). This pattern is consistent with the classical role of Le^x^ as a glycan determinant involved in cell–cell adhesion, leukocyte activation, and inflammatory migration, rather than as a structural tissue marker. Thus, the high frequency of Le^x^ expression in leukocytes observed within lesions suggests that its expression reflects the state of cellular activation and recruitment in the inflammatory microenvironment, which may explain the absence of correlation with the clinical forms of the disease, as demonstrated by the statistical analysis.

The absence of an association between clinical type and degree of expression for Le^x^ and sLe^x^, in contrast to the strong dependence observed on tissue type, reinforces the notion that these epitopes primarily reflect the local functional state, such as endothelial activation and leukocyte recruitment, rather than the clinical classification of the disease.

A particularly relevant aspect was the absence of sLe^x^ expression in the epithelium of sweat gland ducts in lesional areas, contrasting with its presence in non-lesional areas and in control skin. This finding suggests that the chronic inflammation associated with leprosy may interfere with epithelial polarity and the local regulation of glycosylation.

These observations are consistent with previous studies that analyzed the Lewis system from a phenotypic perspective in patients with leprosy, in which specific Lewis phenotypes, particularly Le(a + b−), were associated with susceptibility to the disease, whereas the secretor phenotype was suggested as a protective factor, possibly by interfering with *M. leprae* adhesion to epithelial surfaces [19, 64, 65]. Although the present study focused on the tissue expression of Lewis epitopes rather than systemic phenotypes, these findings reinforce the concept that the Lewis system operates at the interface between host glycosylation and microbial recognition.

Thus, the integration of morphological and statistical findings allows the conclusion that the Le^y^ antigen stands out as the only marker whose expression is significantly associated with the clinical type of leprosy, whereas Le^x^ and sLe^x^ exhibit patterns that are predominantly dependent on tissue type and the local inflammatory state. This interpretation is consistent with reviews emphasizing that the immunopathology of leprosy arises from the complex interplay between host genetic factors and the local inflammatory microenvironment, including molecules involved in cellular recognition and trafficking [2, 10]. This supports a model in which Le^y^ functions not merely as a marker, but as a potential mediator of immune–vascular interactions shaping disease expression.

Some methodological limitations should be highlighted. First, immunohistochemistry provides spatial and semiquantitative information on epitope presence but does not demonstrate direct functionality (e.g., effective binding to DC-SIGN, modulation of intracellular signaling, or in situ angiogenic activity). Second, the use of a polyclonal anti-Mycobacterium tuberculosis antibody, although sensitive, may present cross-reactivity; therefore, confirmation with molecular techniques specific for *M. leprae* (PCR, in situ hybridization) is recommended. Third, the genetic heterogeneity of the population, due to polymorphisms in genes involved in Lewis antigen biosynthesis, such as the Se (FUT2), Le (FUT3), and H (FUT1) genes, influences phenotype, imposing the need for complementary genotyping studies and quantitative glycomic approaches (e.g., lectin-binding assays, mass spectrometry–based glycomics) to strengthen genotype–phenotype correlations.

In this context, direct evaluation of α(1,2)-fucosyltransferase expression would be relevant to further define the regulatory axis underlying Le^y^ expression. Nevertheless, the present study was designed to investigate tissue-level phenotypic patterns rather than the enzymatic control of glycan biosynthesis. This interpretation is biologically supported by evidence that inflammatory signals modulate glycosyltransferase pathways and that α(1,2)-linked fucosylation is enhanced in inflamed endothelial tissues, where it contributes to angiogenesis and leukocyte recruitment [40]. In addition, the central role of fucosylation in immune regulation, cell signaling, and host–pathogen interactions further supports the functional relevance of this pathway in inflammatory contexts [66], consistent with current models of glycan-mediated immune modulation [1, 38, 39, 55].

Based on these findings and the existing literature, we propose specific future directions: (i) *ex vivo/in vitro* functional assays to determine whether Le^y^ expressed on human endothelium facilitates dendritic cell/macrophage adhesion via DC-SIGN; (ii) angiogenesis assays (e.g., tube-formation models) with Le^y^ blocking or masking to evaluate its direct effects on endothelial behavior; (iii) genotypic analysis of the FUT2, FUT3, and H genes in patients to correlate polymorphisms with tissue expression patterns; and (iv) longitudinal pre- and post-treatment studies to assess whether Le^y^ expression regresses with inflammatory resolution, which would help define Le^y^ as a diagnostic marker or a potential therapeutic target.

## Conclusion and future directions

This study demonstrates that the expression of Lewis blood group antigens is dynamically modulated within the inflammatory microenvironment of leprosy lesions. Among the investigated markers, Lewis Y (Le^y^) emerged as the most consistently expressed antigen, displaying broad distribution across endothelial, immune, and neural compartments, and representing the only marker significantly associated with the clinical spectrum of the disease. In contrast, Le^x^ and sLe^x^ exhibited more restricted and tissue-dependent expression patterns, consistent with roles related to leukocyte recruitment and local inflammatory activation rather than disease classification. The combined morphological and statistical evidence supports the interpretation that Le^y^ is closely linked to the immunopathological organization of leprosy lesions. Its preferential endothelial expression, together with its presence in inflammatory and neural structures, is consistent with a role in modulating immune–vascular interactions, leukocyte trafficking, and tissue remodeling. These findings reinforce the concept that glycan-mediated mechanisms contribute to the regulation of host–pathogen interactions in leprosy.

However, given the descriptive nature of immunohistochemical analyses, the functional role of Lewis antigens in these processes remains to be directly demonstrated. Future studies should therefore focus on: (i) functional assays to evaluate whether Le^y^ facilitates immune cell adhesion and signaling through lectin receptors such as DC-SIGN; (ii) experimental models to investigate the role of Le^y^ in endothelial activation and angiogenesis; (iii) genotypic analyses of FUT2, FUT3, and FUT1 to establish genotype–phenotype correlations; and (iv) longitudinal studies to determine whether Le^y^ expression correlates with disease progression or therapeutic response.

Overall, these findings identify Le^y^ as a relevant marker associated with inflammatory organization in leprosy and support further investigation of glycan-mediated pathways as potential targets for biomarker development and immunological intervention.

## Supplementary Information

Below is the link to the electronic supplementary material.

Supplementary Material 1

## Data Availability

All data supporting the findings of this study are available within the article and its Supplementary Information files. The datasets generated and analyzed during the current study, including immunohistochemical scoring, histopathological classifications, and aggregated quantitative results, are presented in the main tables and figures. Additional representative staining patterns are provided in the Supplementary Figures. Further information is available from the corresponding author upon reasonable request.

## References

[1] Kerr-Pontes LR, Barreto ML, Evangelista CM, et al. Socioeconomic, environmental, and behavioural risk factors for leprosy in North-east Brazil: results of a case–control study. Int J Epidemiol. 2006;35:994–1000. 10.1093/ije/dyl072. 16645029 10.1093/ije/dyl072

[2] Grijsen ML, Nguyen TH, Pinheiro RO, et al. Leprosy. Nat Rev Dis Primer. 2024;10:90. 10.1038/s41572-024-00575-1. 10.1038/s41572-024-00575-1 39609422

[3] Scollard DM, Adams LB, Gillis TP, et al. The continuing challenges of leprosy. Clin Microbiol Rev. 2006;19:338–81. 10.1128/CMR.19.2.338-381.2006. 16614253 10.1128/CMR.19.2.338-381.2006 PMC1471987

[4] Britton WJ, Lockwood DN. Lepr Lancet. 2004;363:1209–19. 10.1016/S0140-6736(04)15952-7. 10.1016/S0140-6736(04)15952-7 15081655

[5] Foss NT. Aspectos imunológicos da hanseníase. Med Ribeirão Preto. 1997;30:335–9. 10.11606/issn.2176-7262.v30i3p335-339.

[6] Ridley DS, Jopling WH. Classification of leprosy according to immunity. A five-group system. Int J Lepr Mycobact Dis Off Organ Int Lepr Assoc. 1966;34:255–73. 5950347

[7] CDC. (2025) Clinical Overview of Leprosy. In: Cent. Dis. Control Prev. https://www.cdc.gov/leprosy/hcp/clinical-overview/index.html. Accessed 19 Mar 2026.

[8] Goulart IMB, Penna GO, Cunha G. Imunopatologia da hanseníase: a complexidade dos mecanismos da resposta imune do hospedeiro ao Mycobacterium leprae. Rev Soc Bras Med Trop. 2002;35:363–75. 10.1590/S0037-86822002000400014. 10.1590/s0037-86822002000400014 12170333

[9] Goulart IM, Figueiredo F, Coimbra T, Foss NT. Detection of transforming growth factor-beta 1 in dermal lesions of different clinical forms of leprosy. Am J Pathol. 1996;148:911–7. 8774145 PMC1861719

[10] Fava VM, Dallmann-Sauer M, Schurr E. Genetics of leprosy: today and beyond. Hum Genet. 2020;139:835–46. 10.1007/s00439-019-02087-5. 31713021 10.1007/s00439-019-02087-5

[11] Abel L, Sánchez FO, Oberti J, et al. Susceptibility to leprosy is linked to the human NRAMP1 gene. J Infect Dis. 1998;177:133–45. 10.1086/513830. 9419180 10.1086/513830

[12] Alcaïs A, Alter A, Antoni G, et al. Stepwise replication identifies a low-producing lymphotoxin-alpha allele as a major risk factor for early-onset leprosy. Nat Genet. 2007;39:517–22. 10.1038/ng2000. 17353895 10.1038/ng2000

[13] Ferreira FR, Goulart LR, Silva HD, Goulart IMB. Susceptibility to leprosy may be conditioned by an interaction between the NRAMP1 promoter polymorphisms and the lepromin response. Int J Lepr Mycobact Dis Off Organ Int Lepr Assoc. 2004;72:457–67. 10.1489/1544-581X. (2004)72%253C457:STLMBC%253E2.0.CO;2. 10.1489/1544-581X(2004)72<457:STLMBC>2.0.CO;2 15755200

[14] Jamieson SE, Miller EN, Black GF, et al. Evidence for a cluster of genes on chromosome 17q11-q21 controlling susceptibility to tuberculosis and leprosy in Brazilians. Genes Immun. 2004;5:46–57. 10.1038/sj.gene.6364029. 14735149 10.1038/sj.gene.6364029

[15] Mira MT, Alcaïs A, Van Thuc N, et al. Chromosome 6q25 is linked to susceptibility to leprosy in a Vietnamese population. Nat Genet. 2003;33:412–5. 10.1038/ng1096. 12577057 10.1038/ng1096

[16] Ranque B, Alter A, Mira M, et al. Genomewide linkage analysis of the granulomatous mitsuda reaction implicates chromosomal regions 2q35 and 17q21. J Infect Dis. 2007;196:1248–52. 10.1086/521684. 17955444 10.1086/521684

[17] Tosh K, Meisner S, Siddiqui MR, et al. A region of chromosome 20 is linked to leprosy susceptibility in a South Indian population. J Infect Dis. 2002;186:1190–3. 10.1086/343806. 12355375 10.1086/343806

[18] Miller EN, Jamieson SE, Joberty C, et al. Genome-wide scans for leprosy and tuberculosis susceptibility genes in Brazilians. Genes Immun. 2004;5:63–7. 10.1038/sj.gene.6364031. 14735151 10.1038/sj.gene.6364031

[19] Cooling L. Blood Groups in Infection and Host Susceptibility. Clin Microbiol Rev. 2015;28:801–70. 10.1128/CMR.00109-14. 26085552 10.1128/CMR.00109-14 PMC4475644

[20] Henry SM. Molecular diversity in the biosynthesis of GI tract glycoconjugates. A blood-group-related chart of microorganism receptors. Transfus Clin Biol J Soc Francaise Transfus Sang. 2001;8:226–30. 10.1016/s1246-7820(01)00112-4. 10.1016/s1246-7820(01)00112-4 11499965

[21] Imberty A, Varrot A. Microbial recognition of human cell surface glycoconjugates. Curr Opin Struct Biol. 2008;18:567–76. 10.1016/j.sbi.2008.08.001. 18809496 10.1016/j.sbi.2008.08.001

[22] Hakomori S. Possible functions of tumor-associated carbohydrate antigens. Curr Opin Immunol. 1991;3:646–53. 10.1016/0952-7915(91)90091-e. 1684510 10.1016/0952-7915(91)90091-e

[23] Kuijpers TW. Terminal glycosyltransferase activity: a selective role in cell adhesion. Blood. 1993;81:873–82. 7679005

[24] Henry S, Oriol R, Samuelsson B. Lewis histo-blood group system and associated secretory phenotypes. Vox Sang. 1995;69:166–82. 10.1111/j.1423-0410.1995.tb02591.x. 8578728 10.1111/j.1423-0410.1995.tb02591.x

[25] Kim YS, Yuan M, Itzkowitz SH, et al. Expression of LeY and extended LeY blood group-related antigens in human malignant, premalignant, and nonmalignant colonic tissues. Cancer Res. 1986;46:5985–92. 2428490

[26] Rampelotti J, Bueno E, Valcarenghi D, Geraldo A. Determination of the frequency of Lea Antigens, Leb and Anti-Le antibodies in individuals infected or not by Helicobacter Pylori. J Bacteriol Mycol; 2018.

[27] Ravn V, Dabelsteen E. Tissue distribution of histo-blood group antigens. APMIS Acta Pathol Microbiol Immunol Scand. 2000;108:1–28. 10.1034/j.1600-0463.2000.d01-1.x. 10.1034/j.1600-0463.2000.d01-1.x 10698081

[28] DATASUS. (2000) D.3 Taxa de detecção de hanseníase. http://tabnet.datasus.gov.br/cgi/idb2000/fqd03.htm. Accessed 16 Mar 2026.

[29] Job CK, Chacko CJ. A modification of Fite’s stain for demonstration of M. leprae in tissue sections. Indian J Lepr. 1986;58:17–8. 2427624

[30] Pedal I, Reichert W, Oliveira Corvelo TC. [Seminal vesicle epithelium of Lewis positive individuals secretes Le(a) in sialyl form]. Beitr Gerichtl Med. 1989;47:153–8. 2684147

[31] Pedal I. Blutgruppen-Immunhistochemie: ABO(H)- und Lewis-Antigene menschlicher Gewebe; 16 Tabellen. Stuttgart New York: Thieme; 1987.

[32] Shi S-R, Cote RJ, Taylor CR. Antigen Retrieval Techniques: Current Perspectives. J Histochem Cytochem. 2001;49:931–7. 10.1177/002215540104900801. 11457921 10.1177/002215540104900801

[33] Ramos-Vara JA, Miller MA. When Tissue Antigens and Antibodies Get Along: Revisiting the Technical Aspects of Immunohistochemistry—The Red, Brown, and Blue Technique. Vet Pathol. 2014;51:42–87. 10.1177/0300985813505879. 24129895 10.1177/0300985813505879

[34] Meyerholz DK, Beck AP. Principles and approaches for reproducible scoring of tissue stains in research. Lab Invest. 2018;98:844–55. 10.1038/s41374-018-0057-0. 29849125 10.1038/s41374-018-0057-0

[35] Hunter JD. Matplotlib: A 2D Graphics Environment. Comput Sci Eng. 2007;9:90–5. 10.1109/MCSE.2007.55.

[36] Virtanen P, Gommers R, Oliphant TE, et al. SciPy 1.0: fundamental algorithms for scientific computing in Python. Nat Methods. 2020;17:261–72. 10.1038/s41592-019-0686-2. 32015543 10.1038/s41592-019-0686-2 PMC7056644

[37] Harris CR, Millman KJ, van der Walt SJ, et al. Array programming with NumPy. Nature. 2020;585:357–62. 10.1038/s41586-020-2649-2. 32939066 10.1038/s41586-020-2649-2 PMC7759461

[38] Waskom M. seaborn: statistical data visualization. J Open Source Softw. 2021;6:3021. 10.21105/joss.03021.

[39] Groux-Degroote S, Cavdarli S, Uchimura K, et al. Glycosylation changes in inflammatory diseases. Advances in Protein Chemistry and Structural Biology. Elsevier; 2020. pp. 111–56. 10.1016/bs.apcsb.2019.08.008 31997767

[40] Isozaki T, Amin MA, Ruth JH, et al. Fucosyltransferase 1 mediates angiogenesis in rheumatoid arthritis. Arthritis Rheumatol. 2014;66:2047–58. 10.1002/art.38648. 24692243 10.1002/art.38648 PMC4426876

[41] Baek H, Yang S-W, Kim S, et al. Development of Anti-Inflammatory Agents Utilizing DC-SIGN Mediated IL-10 Secretion in Autoimmune and Immune-Mediated Disorders: Bridging Veterinary and Human Health. Int J Mol Sci. 2025;26:2329. 10.3390/ijms26052329. 40076949 10.3390/ijms26052329 PMC11901132

[42] Zheng RB, Jégouzo SAF, Joe M, et al. Insights into Interactions of Mycobacteria with the Host Innate Immune System from a Novel Array of Synthetic Mycobacterial Glycans. ACS Chem Biol. 2017;12:2990–3002. 10.1021/acschembio.7b00797. 29048873 10.1021/acschembio.7b00797 PMC5735379

[43] Leano HADM, Araújo KMDFA, Bueno IDC, et al. Socioeconomic factors related to leprosy: an integrative literature review. Rev Bras Enferm. 2019;72:1405–15. 10.1590/0034-7167-2017-0651. 31531668 10.1590/0034-7167-2017-0651

[44] Lázaro FP, Werneck RI, Mackert CCO, et al. A major gene controls leprosy susceptibility in a hyperendemic isolated population from north of Brazil. J Infect Dis. 2010;201:1598–605. 10.1086/652007. 20388034 10.1086/652007

[45] De Oliveira Mackert CC, Lázaro FP, Olandowski M, et al. Insights into leprosy epidemiology from an isolated population located in the Brazilian Amazon. Sci Rep. 2025;15:6103. 10.1038/s41598-025-90399-0. 39971783 10.1038/s41598-025-90399-0 PMC11840054

[46] Rabinovich GA, Croci DO. Regulatory Circuits Mediated by Lectin-Glycan Interactions in Autoimmunity and Cancer. Immunity. 2012;36:322–35. 10.1016/j.immuni.2012.03.004. 22444630 10.1016/j.immuni.2012.03.004

[47] Malý P, Thall A, Petryniak B, et al. The alpha(1,3)fucosyltransferase Fuc-TVII controls leukocyte trafficking through an essential role in L-, E-, and P-selectin ligand biosynthesis. Cell. 1996;86:643–53. 10.1016/s0092-8674(00)80137-3. 8752218 10.1016/s0092-8674(00)80137-3

[48] Halloran MM, Carley WW, Polverini PJ et al. (2000) Ley/H: an endothelial-selective, cytokine-inducible, angiogenic mediator. J Immunol Baltim Md 1950 164:4868–4877. 10.4049/jimmunol.164.9.4868 10.4049/jimmunol.164.9.4868 10779796

[49] Zhu K, Amin MA, Kim MJ, et al. A novel function for a glucose analog of blood group H antigen as a mediator of leukocyte-endothelial adhesion via intracellular adhesion molecule 1. J Biol Chem. 2003;278:21869–77. 10.1074/jbc.M213052200. 12672794 10.1074/jbc.M213052200

[50] Wipplinger M, Mink S, Bublitz M, Gassner C. Regulation of the Lewis Blood Group Antigen Expression: A Literature Review Supplemented with Computational Analysis. Transfus Med Hemotherapy. 2024;51:225–36. 10.1159/000538863. 10.1159/000538863 PMC11318966 39135855

[51] Kelly RJ, Rouquier S, Giorgi D, et al. Sequence and expression of a candidate for the human Secretor blood group alpha(1,2)fucosyltransferase gene (FUT2). Homozygosity for an enzyme-inactivating nonsense mutation commonly correlates with the non-secretor phenotype. J Biol Chem. 1995;270:4640–9. 10.1074/jbc.270.9.4640. 7876235 10.1074/jbc.270.9.4640

[52] Marionneau S, Cailleau-Thomas A, Rocher J, et al. ABH and Lewis histo-blood group antigens, a model for the meaning of oligosaccharide diversity in the face of a changing world. Biochimie. 2001;83:565–73. 10.1016/s0300-9084(01)01321-9. 11522384 10.1016/s0300-9084(01)01321-9

[53] Geijtenbeek TBH, Van Vliet SJ, Koppel EA, et al. Mycobacteria target DC-SIGN to suppress dendritic cell function. J Exp Med. 2003;197:7–17. 10.1084/jem.20021229. 12515809 10.1084/jem.20021229 PMC2193797

[54] Gringhuis SI, den Dunnen J, Litjens M, et al. C-type lectin DC-SIGN modulates Toll-like receptor signaling via Raf-1 kinase-dependent acetylation of transcription factor NF-kappaB. Immunity. 2007;26:605–16. 10.1016/j.immuni.2007.03.012. 17462920 10.1016/j.immuni.2007.03.012

[55] Appelmelk BJ, van Die I, van Vliet SJ, et al. Cutting edge: carbohydrate profiling identifies new pathogens that interact with dendritic cell-specific ICAM-3-grabbing nonintegrin on dendritic cells. J Immunol Baltim Md 1950. 2003;170:1635–9. 10.4049/jimmunol.170.4.1635. 10.4049/jimmunol.170.4.1635 12574325

[56] Shi C-S, Shi G-Y, Hsiao H-M, et al. Lectin-like domain of thrombomodulin binds to its specific ligand Lewis Y antigen and neutralizes lipopolysaccharide-induced inflammatory response. Blood. 2008;112:3661–70. 10.1182/blood-2008-03-142760. 18711002 10.1182/blood-2008-03-142760 PMC2572793

[57] Pinho SS, Reis CA. Glycosylation in cancer: mechanisms and clinical implications. Nat Rev Cancer. 2015;15:540–55. 10.1038/nrc3982. 26289314 10.1038/nrc3982

[58] Madigan CA, Cambier CJ, Kelly-Scumpia KM, et al. A Macrophage Response to Mycobacterium leprae Phenolic Glycolipid Initiates Nerve Damage in Leprosy. Cell. 2017;170:973–e98510. 10.1016/j.cell.2017.07.030. 28841420 10.1016/j.cell.2017.07.030 PMC5848073

[59] Serrano-Coll H, Salazar-Peláez L, Acevedo-Saenz L, Cardona-Castro N. Mycobacterium leprae-induced nerve damage: direct and indirect mechanisms. Pathog Dis. 2018;76. 10.1093/femspd/fty062. 10.1093/femspd/fty062 30052986

[60] Walker SL, Lockwood DNJ. Leprosy type 1 (reversal) reactions and their management. Lepr Rev. 2008;79:372–86. 19274984

[61] Varki A. Biological roles of glycans. Glycobiology. 2017;27:3–49. 10.1093/glycob/cww086. 27558841 10.1093/glycob/cww086 PMC5884436

[62] Ley K, Laudanna C, Cybulsky MI, Nourshargh S. Getting to the site of inflammation: the leukocyte adhesion cascade updated. Nat Rev Immunol. 2007;7:678–89. 10.1038/nri2156. 17717539 10.1038/nri2156

[63] Stocks SC, Kerr MA. Stimulation of neutrophil adhesion by antibodies recognizing CD15 (LeX) and CD15-expressing carcinoembryonic antigen-related glycoprotein NCA-160. Biochem J. 1992;288:23–7. 10.1042/bj2880023. 1359882 10.1042/bj2880023 PMC1132074

[64] Naafs B, Silva E, Vilani-Moreno F, et al. Factors influencing the development of leprosy: an overview. Int J Lepr Mycobact Dis Off Organ Int Lepr Assoc. 2001;69:26–33. 11480313

[65] Silva EA, Rúbio EM, Ura S. Sistema sangüíneo Lewis em pacientes hansenianos. Hansen Int Hansen E Outras Doenças Infecc. 2000;25:115–20. 10.47878/hi.2000.v25.36433.

[66] Zhang N-Z, Zhao L-F, Zhang Q, et al. Core fucosylation and its roles in gastrointestinal glycoimmunology. World J Gastrointest Oncol. 2023;15:1119–34. 10.4251/wjgo.v15.i7.1119. 37546555 10.4251/wjgo.v15.i7.1119 PMC10401475

