# Supplementary material for: Lewis Y (Ley) orchestrates leukocyte trafficking and inflammatory remodeling in leprosy patients from the Brazilian Amazon
Source: Immunol Res. 2026 Apr 9;74(1):38. doi: 10.1007/s12026-026-09776-0 (PMC13065620; doi:10.1007/s12026-026-09776-0)
Supplement: Supplementary file 1 — Supplementary Material 1 [file 12026_2026_9776_MOESM1_ESM.docx]

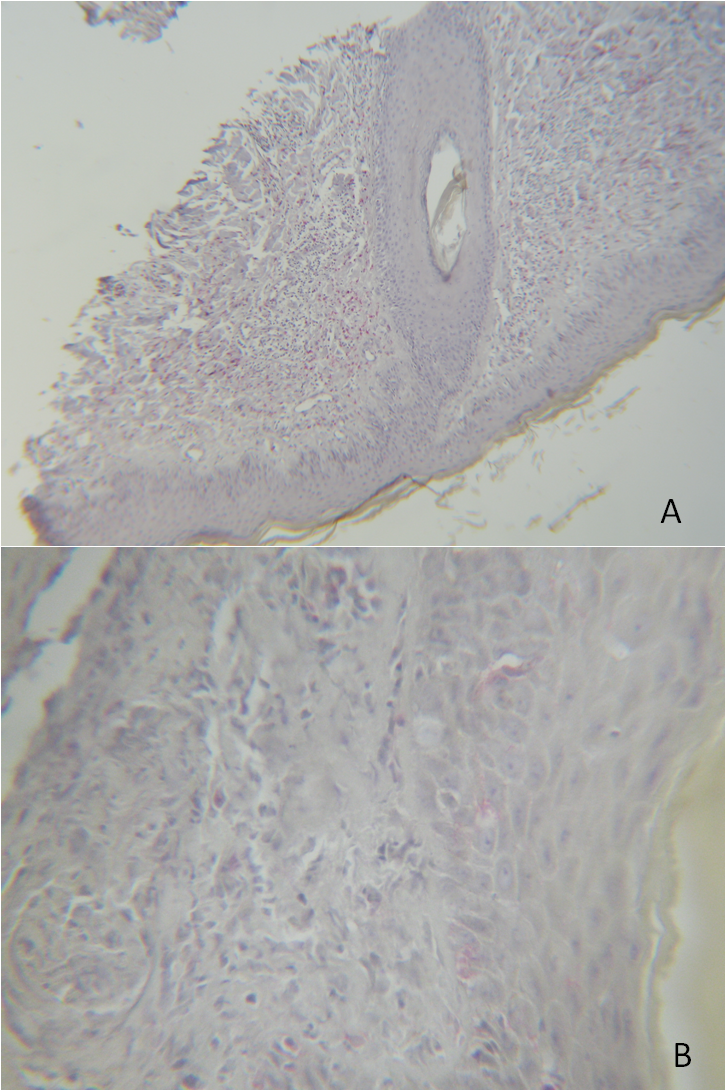


**Supplementary Figure S1. Expression pattern of Lewis X (Lex) antigen in epidermal structures.** (A) Normal skin showing absent or minimal Lex immunoreactivity. (B) Skin fragment from a patient with leprosy (TT) showing focal and low-intensity Lex staining in epidermal cells. Immunohistochemistry was performed using an alkaline phosphatase-based detection system. Counterstain: hematoxylin. Magnification: 100×.


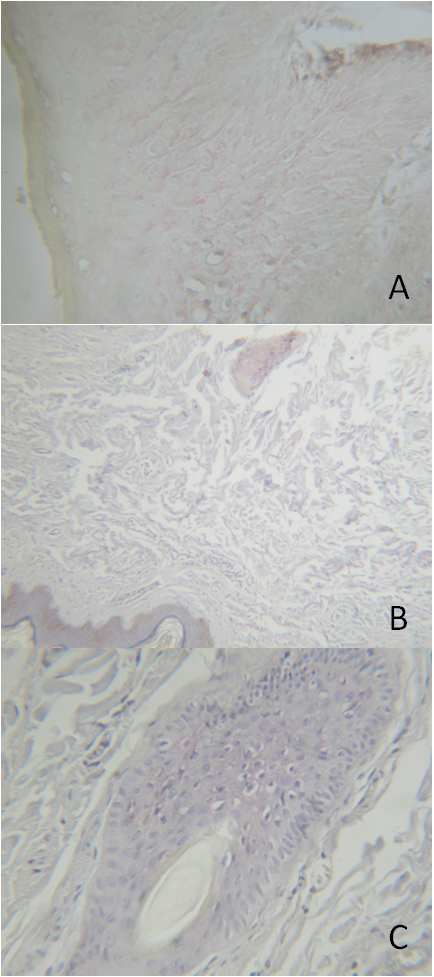


**Supplementary Figure S2. Expression pattern of sialyl-Lewis X (sLex) antigen in epidermal and dermal structures.** (A) Normal skin showing epithelial reactivity in the spinous layer. (B) Dermal region from normal skin with no significant staining. (C) Skin fragment from a patient with leprosy (IN) showing variable sLex expression in epidermal and dermal compartments. Immunohistochemistry was performed using an alkaline phosphatase-based detection system. Counterstain: hematoxylin. Magnification: 400×.
